# Diffusion Tensor Metrics as Biomarkers in Alzheimer's Disease

**DOI:** 10.1371/journal.pone.0049072

**Published:** 2012-11-07

**Authors:** Julio Acosta-Cabronero, Stephanie Alley, Guy B. Williams, George Pengas, Peter J. Nestor

**Affiliations:** 1 Cognition, Memory and Language Group, Neurology Unit, Department of Clinical Neurosciences, School of Clinical Medicine, University of Cambridge, Cambridge, United Kingdom; 2 Department of Applied Mathematics and Theoretical Physics, Centre for Mathematical Sciences, University of Cambridge, Cambridge, United Kingdom; 3 Wolfson Brain Imaging Centre, Department of Clinical Neurosciences, School of Clinical Medicine, University of Cambridge, Addenbrooke’s Hospital, Cambridge, United Kingdom; 4 German Center for Neurodegenerative Diseases (DZNE), Magdeburg, Germany; University of Maryland, College Park, United States of America

## Abstract

**Background:**

Although diffusion tensor imaging has been a major research focus for Alzheimer’s disease in recent years, it remains unclear whether it has sufficient stability to have biomarker potential. To date, frequently inconsistent results have been reported, though lack of standardisation in acquisition and analysis make such discrepancies difficult to interpret. There is also, at present, little knowledge of how the biometric properties of diffusion tensor imaging might evolve in the course of Alzheimer’s disease.

**Methods:**

The biomarker question was addressed in this study by adopting a standardised protocol both for the whole brain (tract-based spatial statistics), and for a region of interest: the midline corpus callosum. In order to study the evolution of tensor changes, cross-sectional data from very mild (N = 21) and mild (N = 22) Alzheimer’s disease patients were examined as well as a longitudinal cohort (N = 16) that had been rescanned at 12 months.

**Findings and Significance:**

The results revealed that increased axial and mean diffusivity are the first abnormalities to occur and that the first region to develop such significant differences was mesial parietal/splenial white matter; these metrics, however, remained relatively static with advancing disease indicating they are suitable as ‘state-specific’ markers. In contrast, increased radial diffusivity, and therefore decreased fractional anisotropy–though less detectable early–became increasingly abnormal with disease progression, and, in the splenium of the corpus callosum, correlated significantly with dementia severity; these metrics therefore appear ‘stage-specific’ and would be ideal for monitoring disease progression. In addition, the cross-sectional and longitudinal analyses showed that the progressive abnormalities in radial diffusivity and fractional anisotropy always occurred in areas that had first shown an increase in axial and mean diffusivity. Given that the former two metrics correlate with dementia severity, but the latter two did not, it would appear that increased axial diffusivity represents an upstream event that precedes neuronal loss.

## Introduction

There is presently considerable interest in trying to expedite therapeutic development through the use of biomarkers to track change in Alzheimer’s disease. To date, most biomarker work with magnetic resonance imaging (MRI) has focused on structural acquisitions to measure atrophy [1]. A potential strength of MRI, compared, for instance, to nuclear medicine imaging techniques, is that multiple types of data–offering complimentary information–can be acquired in a single scanning session. Diffusion tensor imaging (DTI) is one such method that offers information about white matter integrity. Early work using DTI in Alzheimer’s disease focused particularly on fractional anisotropy or FA [2], [3], [4], though some studies have identified that this measure is insensitive to early white matter disruption in Alzheimer’s disease [5], [6]. This is because both axial (λ_1_) and radial diffusivity (RD) increase and therefore FA, which is a function of the ratio of these two measures, can remain relatively unperturbed. Conflicting results, however, have been reported in Alzheimer’s disease; some studies show emphatic mean diffusivity (MD) differences, believed to be largely driven by λ_1_ alterations [5], [7], [8]; some report stronger RD effects [7], [9], [10], [11]–the former in limbic tracts only; and some show, in addition, highly-abnormal FA behaviours [2], [4], [7], [10], [12], [13], [14]. While these findings have helped define the landscape of diffusion changes in Alzheimer’s disease, it is unclear how the various tensor metrics evolve over time, and, whether better understanding of such evolution may explain some of these apparently conflicting results. In order to address this issue, we used a common acquisition protocol to examine the evolution of tensor changes over the course of Alzheimer’s disease by studying both cross-sectional data at differing dementia severities and longitudinal change over a 12 month period. We performed whole-brain analyses and also assessed a directly-visualised white matter tract that is known to be severely damaged in Alzheimer’s disease *i.e.* the corpus callosum [3], [4], [7], [8], [11], [12], [13], [15], [16]. The aim was to identify whether the various tensor metrics might show differential preference as biomarkers to track change or for early diagnosis, and if so, which are the best for each purpose.

**Table 1:** Demographic summary including cognitive features for Alzheimer’s disease patients and for a group of elderly controls.

		Control (N = 26)	Alzheimer’s Disease (N = 43)	Very mild Alzheimer’s disease (N = 21)	Mild Alzheimer’s disease (N = 22)
**General Demographics**	**Gender, M:F**	11∶15	26∶17	13∶8	13∶9
	**Age at imaging, years**	68 (6)	70 (6)	72 (5)	69 (6)
**Global Cognition**	**MMSE/30**	29.1 (0.8)	23.7 (3.6)**	25.9 (1.6)**	21.7 (3.9)**∧
	**ACE-R/100**	94.6 (3.0)	71.5 (11.9)**	81.4 (4.0)**	62.1 (8.8)**∧
**ACE-R Subscores**	**Attention & Orientation/18**	17.9 (0.3)	15.4 (2.7)**	17.0 (1.4)*	13.9 (2.8)**∧
	**Memory/26**	24.5 (1.9)	10.9 (4.3)**	13.7 (4.1)**	8.2 (2.4)**∧
	**Fluency/14**	12.1 (1.7)	8.3 (3.2)**	10.6 (1.9)*	6.2 (2.6)**∧
	**Language/26**	24.9 (0.9)	23.0 (2.6)**	24.6 (1.2)	21.5 (2.7)**∧
	**Visuospatial/16**	15.4 (0.9)	13.7 (2.8)**	15.1 (1.2)	12.3 (3.1)**∧

Disease severity (as measured by ACE-R) enabled a median split of the patient cohort into very mild and mild Alzheimer’s disease subgroups.

Where appropriate, group values are given as mean (SD).

MMSE/30 = Mini-mental state examination score out of 30-point total; ACE-R/100 = Addenbrooke’s cognitive examination-revised score out of 100-point total.

Wilcoxon rank-sum significance levels: *P<0.01 (Alzheimer’s disease worse than controls, two-tailed); **P<0.05 (Alzheimer’s disease worse than controls, two-tailed, Bonferroni-corrected on n = 28 tests); ∧P<0.05 (Mild worse than very mild Alzheimer’s disease, two-tailed, Bonferroni-corrected on n = 28).

## Methods

### Ethics Statement

The study was approved by the Cambridgeshire 2 Research Ethics Committee (Reference: 07/H0308/215) and by the National Hospital for Neurology and Neurosurgery & Institute of Neurology Joint Research Ethics Committee (Reference: 05/Q0512/12).

Written informed consent was obtained from all the participants. In the context of this study, although the target population were patients suffering from a degenerative brain condition, we expected them to have capacity to consent as only the mild stages of Alzheimer’s disease were studied. Before inclusion, every patient was assessed by an expert cognitive neurologist to ensure capacity and this was indeed the case. Although we had ethical permission to scan patients who lacked capacity (with caregiver consent), this was not needed in the present study.

**Figure 1:**
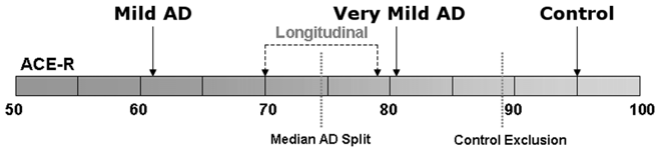
Cognitive status. Depiction of average cognitive profiles for all subject cohorts assessed in this study.

**Table 2:** Cognitive profile evolution of a group of early-stage Alzheimer’s disease patients that was followed-up for a period of 12 months.

Alzheimer’s Disease(Longitudinal, N = 16)		Baseline	12 Months
**General** **Demographics**	**Gender, M:F**	8∶8	
**Global Cognition**	**MMSE/30**	25.1 (2.0)	22.6 (4.9)^§^
	**ACE-R/100**	77.5 (7.3)	70.3 (13.6)**
**ACE-R Subscores**	**Attention &** **Orientation/18**	16.1 (1.1)	14.8 (3.5)
	**Memory/26**	13.5 (3.9)	9.8 (3.4)**
	**Fluency/14**	9.6 (2.5)	8.5 (3.8)
	**Language/26**	23.6 (1.8)	23.1 (2.5)
	**Visuospatial/16**	14.7 (1.5)	14.0 (2.9)

Cognitive measures are given as mean (SD).

MMSE/30 = Mini-mental state examination score out of 30-point total; ACE-R/100 = Addenbrooke’s cognitive examination-revised score out of 100-point total.

Wilcoxon signed-rank significance levels: ^§^0.01<P<0.05 (Alzheimer’s disease: 12 months worse than baseline, two-tailed); **P<0.05 (Alzheimer’s disease: 12 months worse than baseline, two-tailed, Bonferroni-corrected on n = 7).

### Subjects

#### Cross-sectional cohorts

Forty-three patients with early-stage probable Alzheimer’s disease according to criteria from the National Institute of Neurological and Communicative Disorders and Stroke and the Alzheimer’s Disease and Related Disorders Association (NINCDS-ADRDA) [17] were recruited from the memory clinic at Addenbrooke’s Hospital (Cambridge, UK). For cross-sectional comparisons, 26 matched controls were also recruited and were screened to exclude neurological or major psychiatric illness. They performed normally on cognitive screening: mini-mental state examination or MMSE [18] and Addenbrooke’s cognitive examination–revised or ACE-R [19]. The control exclusion criteria was ACE-R<88 (out of 100). Note that 25 patients (ACE-R = 69.5±12.5) and 12 elderly controls previously assessed [5] were also included in the present study.

Enabled by an ACE-R median split, patients were further subcategorised according to disease severity into very mild (best 50% ACE-R) and mild Alzheimer’s disease cohorts (worst 50% ACE-R) (Table 1). The very mild patient group (N = 21) included 16 subjects who were diagnosed with the mild-cognitive impairment stage of Alzheimer’s disease–*i.e.* these were patients scanned with mild-cognitive impairment that were subsequently shown to have probable Alzheimer’s disease by confirming progressive cognitive decline with longitudinal follow-up. Although significantly impaired overall on both global cognitive measures, the very mild Alzheimer’s disease group was unimpaired on language and visuospatial subsections of the ACE-R at the time of scanning. The mild Alzheimer’s disease group, however, was impaired in all subdomains of the ACE-R compared both to controls and to the very mild group of patients.

**Figure 2:**
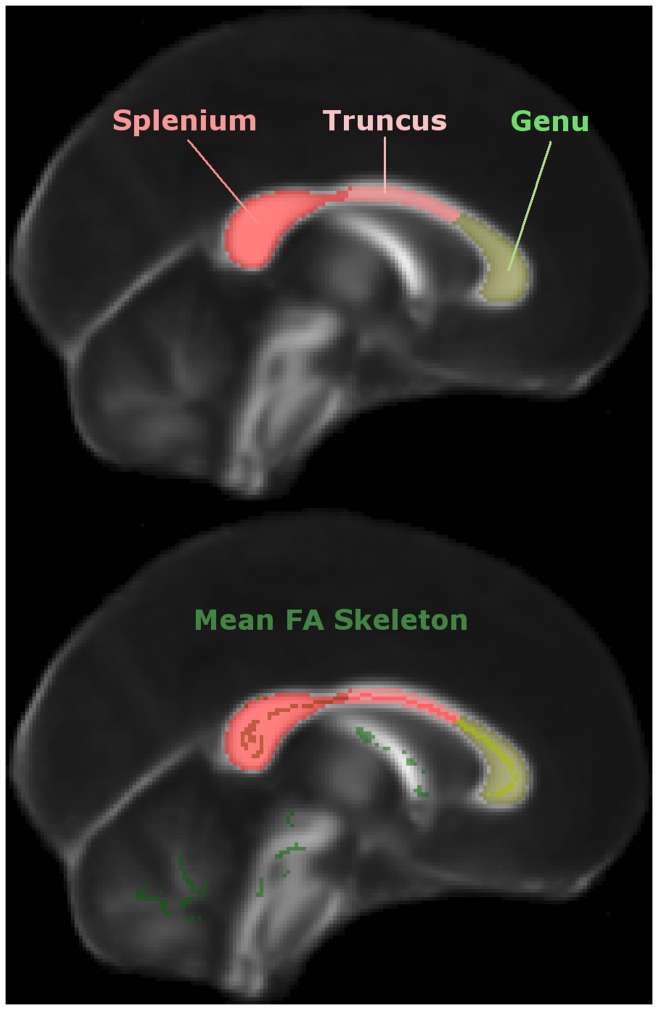
Corpus callosum subdivision. Depiction of the semi-automated callosal subdivision into splenium, truncus and genu (top), and their intersection with the mean FA skeleton inferred from N = 69 subjects–N = 43 Alzheimer’s disease patients and N = 26 matched controls (bottom).

#### Longitudinal cohort

A subgroup of 16 Alzheimer’s disease subjects –9 of which were diagnosed with mild cognitive impairment at baseline – was followed-up with scans and neuropsychology tests taking place 12 months apart. Serendipitously, the cognitive scores shown in Table 2 revealed a similar transition from baseline to 12 months as was seen in the cross-sectional data on the very mild Alzheimer’s disease stage relative to controls (Table 1)–the memory subdomain of the ACE-R was also the most impaired, followed by statistical trends towards fluency and attention/orientation deficits; whereas in contrast, language and visuospatial abilities, despite being slightly reduced, remained comparatively preserved. Group-average cognitive profiles are summarised in Figure 1.

**Figure 3:**
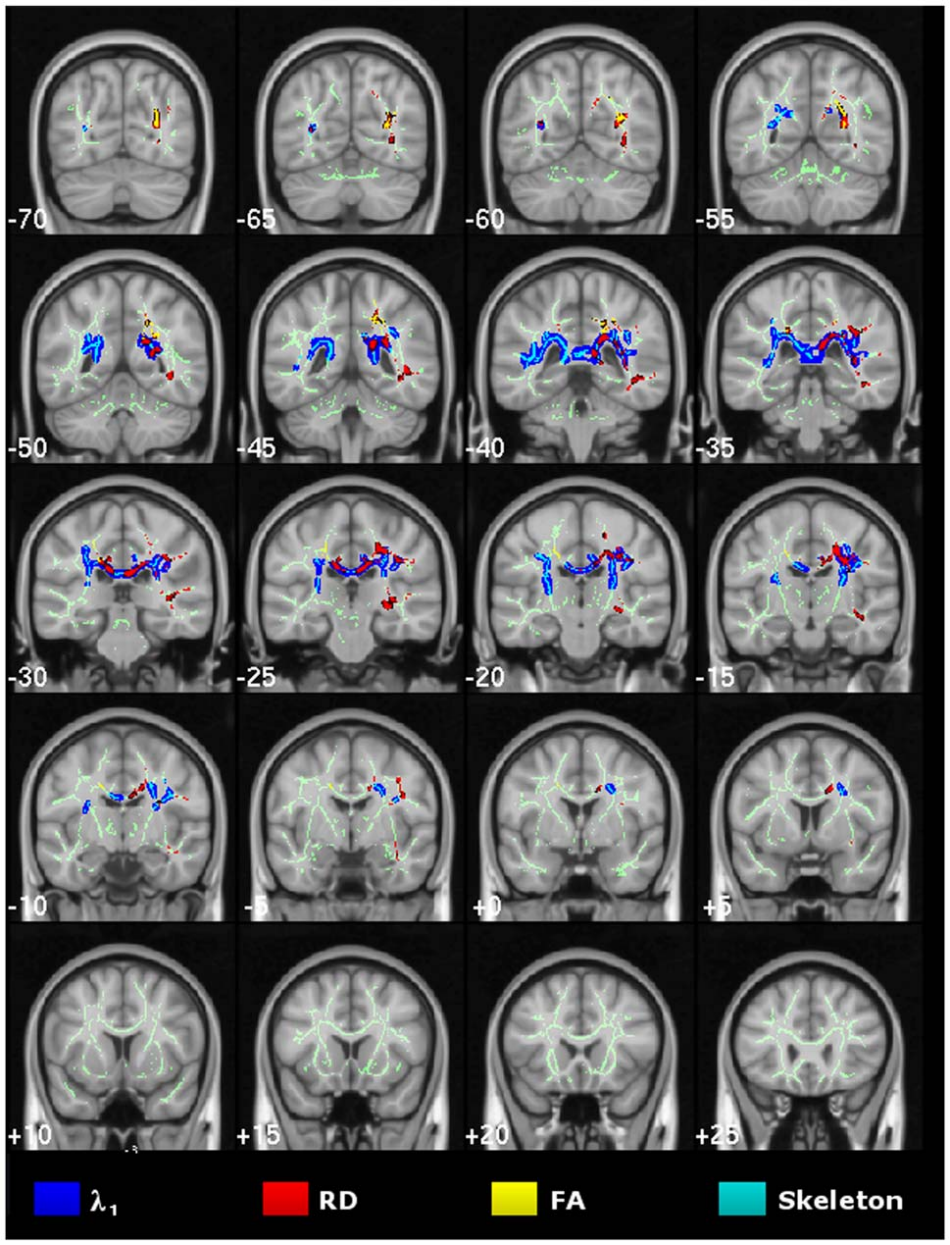
Cross-sectional study of very mild Alzheimer’s disease. TBSS results for the very mild Alzheimer’s disease group compared to controls. Statistical maps (thresholded at TFCE-P<0.05) for increased axial/radial diffusivity and reduced FA overlaid onto the mean FA skeleton and the MNI152 template. Coronal depths are given in millimetres.

### Imaging

All patients were scanned within an average of 1.3 months (standard deviation = 1.9 months) from a cognitive assessment. MRI scans were performed on a Siemens Trio 3T system (Siemens Medical Systems, Erlangen, Germany) with gradient coils capable of 45 mT/m and 200 T/m/s slew rate. A standard 12-channel phased-array total imaging matrix head-coil (Siemens Medical Systems, Erlangen, Germany) was used to transmit/receive radio-frequency signals.

#### Diffusion tensor imaging

Diffusion datasets were acquired using a twice-refocused, single-shot, echo-planar imaging pulse sequence [20]: repetition time (TR)/echo time (TE)/number of excitations = 7800 ms/90 ms/1; matrix, 96×96; 63 contiguous axial slices; isotropic voxel resolution of 2×2×2 mm^3^; bandwidth of 1628 Hz/pixel and echo spacing of 0.72 ms. The tensor was computed using 63 non-collinear diffusion directions (*b* = 1000 s/mm^2^) that were maximally spread by considering the minimal energy arrangement of point charges on a sphere, and one scan without diffusion weighting (*b* = 0 s/mm^2^, *b_0_*). We allowed for parallel acquisition of independently-reconstructed images using generalised, autocalibrating, partially-parallel acquisitions or GRAPPA [21]; acceleration factor of 2 and 39 reference lines. The total scan time was 8′44′’.

**Figure 4:**
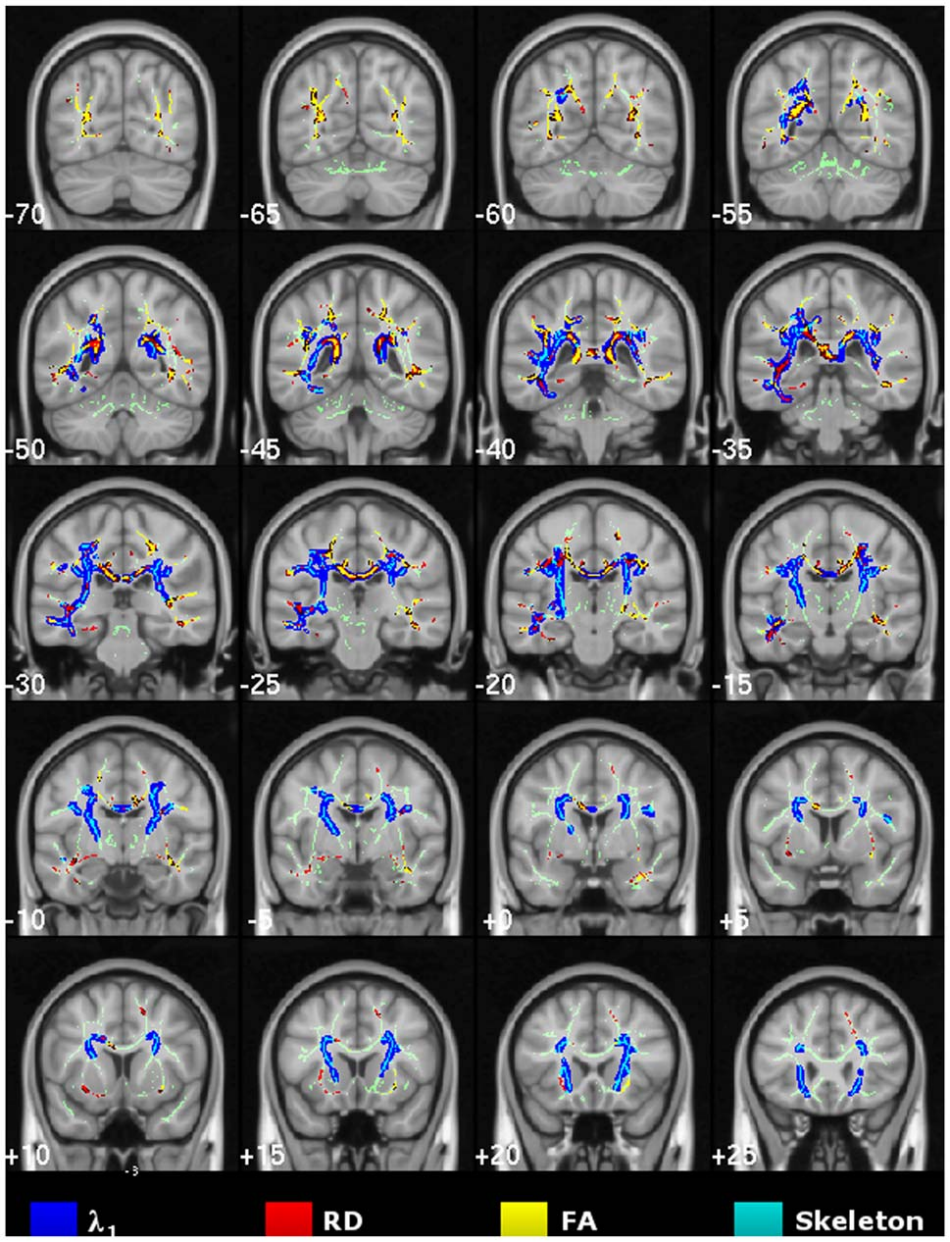
Cross-sectional study of mild Alzheimer’s disease. TBSS results for the mild-stage Alzheimer’s disease group compared to controls. Thresholded (TFCE-P<0.05) statistical maps for increased axial/radial diffusivity and reduced FA were overlaid onto the mean FA skeleton and the MNI152 template. Coronal depths are given in millimetres.

#### Volumetric T_1_ imaging

T_1_-weighted anatomical images were also acquired in the same session. The structural scan consisted of 3D magnetisation-prepared, rapid gradient-echo (MPRAGE) volumes with the following imaging parameters: TR/TE/inversion time/flip angle = 2300 ms/2.86 ms/900 ms/9°, 144 slices, 192×192 matrix dimensions and 1.25×1.25×1.25 mm^3^ voxel size. Receiver bandwidth and echo spacing were 240 Hz/pixel and 6.7 ms, respectively. The total scan time was 7′23′’.

#### Ultrafast T_2_ imaging

Whole-brain, T_2_-weighted, half-Fourier acquisition, single-shot turbo spin echo (HASTE) images were acquired to ensure that vascular pathology was not significant in any subject. The following scan parameters were used: TR/TE/flip angle/turbo factor = 2000 ms/89 ms/150°/205; matrix, 320×256; 25 axial slices (distance factor: 20%); voxel resolution, 0.7×0.9×4 mm^3^; 5/8-phase partial Fourier transform; bandwidth and echo spacing of 401 Hz/pixel and 5.58 ms, respectively. GRAPPA mode was enabled with an acceleration factor of 2 and 24 reference lines, resulting in a total scan time of 52 seconds.

In all acquisitions the field of view was aligned in stereotactic space: the axial plane was aligned to the anterior commissure–posterior commissure line, and the sagittal plane to the inter-hemispheric fissure. In addition to stereotactic alignment, in order to maximise acquisition consistency across subjects, the scanning bed was adjusted to co-localise the centre of the thalamus in the mid-sagittal plane with the scanner isocentre.

**Figure 5:**
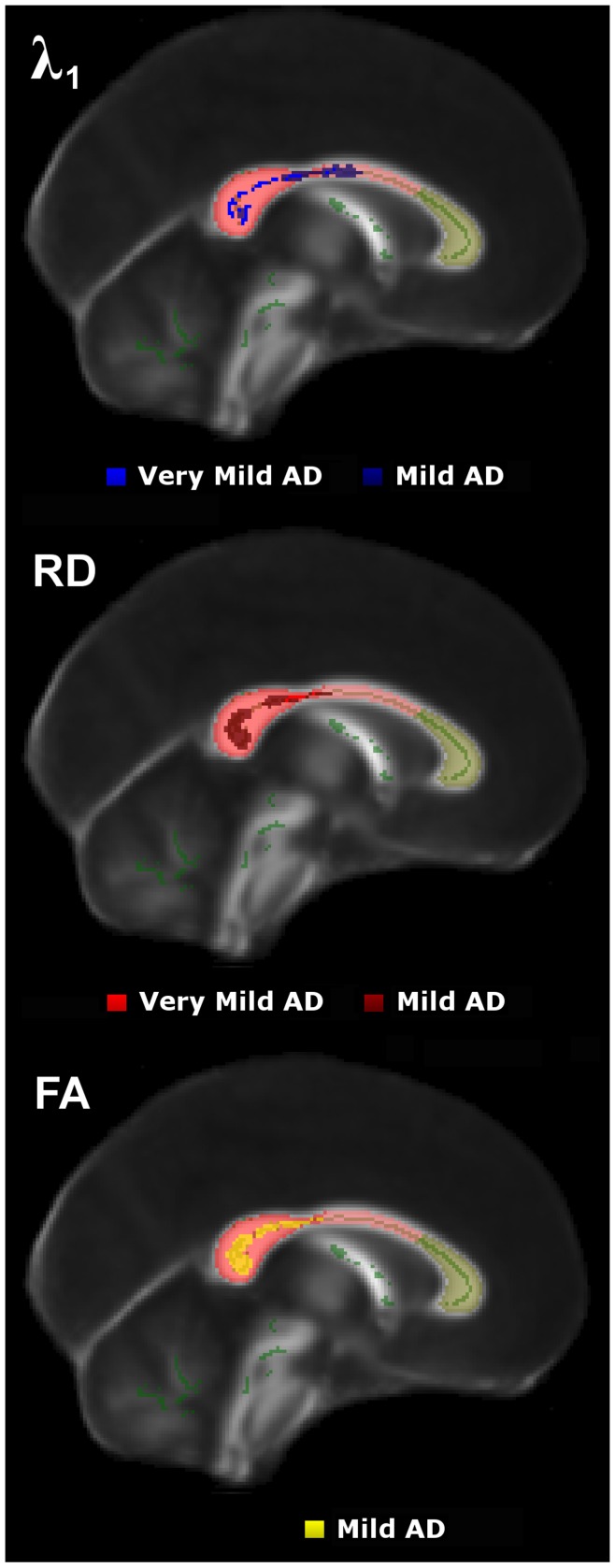
Cross-sectional results in the mid-sagittal corpus callosum. TBSS results across the sagittal midline for very mild and mild Alzheimer’s disease groups compared to controls.

### Data Processing and Analysis

#### Diffusion tensor parametric maps

The Oxford Centre for Functional MRI of the Brain (FMRIB) software library (FSL v4.1.2) [22] was used to correct for motion and eddy currents, fit the diffusion tensor and compute axial, radial and mean diffusivity as well as fractional anisotropy whole-brain maps. Initially, each diffusion-weighted volume was affine-aligned to its corresponding *b_0_* image using the FMRIB’s linear image registration tool (FLIRT v5.4.2) [23]; this pre-processing step corrects for motion artefacts and eddy-current distortions. In addition, in order to eliminate spurious voxels, brain masks of each *b_0_* image were generated using the brain-extraction tool (BET v2.1) [24] with fractional threshold, *f* = 0.1, and vertical gradient, *g* = 0. The FMRIB’s diffusion toolbox (FDT v2.0) was then used to fit the tensor and compute the diagonal elements (λ_1_, λ_2_ and λ_3_) at each brain voxel, from which the derived metrics RD, MD and FA were also inferred. Note that negative primary eigenvalues were deemed unphysical and were set to 0–a visual inspection of the spatial distribution of negative eigenvalues revealed that they were located in the periphery of white matter bundles *i.e.* adjacent to other tissue types and far from tract centres.

#### Tract-based spatial statistics (TBSS) analysis

The TBSS approach [25] was used to perform whole-brain statistical analyses at white matter tract centres. Spatial normalisation was achieved by warping all FA images to the 1×1×1 mm^3^ FMRIB58_FA standard template (FMRIB, University of Oxford, UK) in MNI152 space (Montreal Neurological Institute, McGill University, Canada) using the FMRIB's non-linear image registration tool (FNIRT v1.0). All–patients (N = 43) and controls (N = 26)–warped FA maps were averaged to create the mean FA template, from which the mean FA skeleton was derived (FA>0.2). Finally, all subjects’ spatially normalised FA, λ_1_, RD and MD data were projected onto the skeleton and fed into voxel-wise statistics, where 10,000 permutations of the data were generated using randomise v2.1 with threshold-free cluster enhancement (TFCE) enabled [26]. The following statistical comparisons were made: (i) cross-sectional: very mild (N = 21) and mild (N = 22) Alzheimer’s disease patients versus controls (N = 26); and (ii) longitudinal: Alzheimer’s disease (N = 16) at 12 months versus baseline data. Note that for subgroup comparisons–*i.e.* very mild and mild Alzheimer’s disease against controls–and for the longitudinal assessment, we used the mean FA skeleton derived from all 69 subjects to compute the skeletonisation vectors. All statistical maps were thresholded at a TFCE level of P<0.05 to help prevent the known issue of Type I errors in voxel-wise experiments.

**Table 3:** Alzheimer’s disease skeletonised DTI parametric comparisons in different mid-sagittal callosal areas.

Alzheimer’s disease (N = 43) vs. Control (N = 26)	Splenium	Truncus	Genu
**λ_1_**	−2.24^§^	−1.39	−0.22
**RD**	−3.08**	−0.76	0.53
**MD**	−3.39**	−1.08	0.30
**FA**	2.45^§^	0.40	−1.18

Results are given as Wilcoxon rank-sum Z-statistic.

Significance levels: ^§^0.01<P<0.05 (Alzheimer’s disease worse than controls, two-tailed); **P<0.05 (Alzheimer’s disease worse than controls, two-tailed, Bonferroni-corrected on n = 12).

#### Regional analysis

Region-of-interest analyses can be confounded in DTI studies where tracts are not directly visualised such as in the white matter of the cerebral hemispheres. For instance, the effect of atrophy in a patient group could mean that regions of interest are subtly–though systematically–misplaced with respect to tract anatomy, and this could cause spurious alterations in tensor metrics. Furthermore, the impact of crossing fibres can make changes in tensor behaviour difficult to interpret. For instance, increased λ_1_ has been previously reported in Alzheimer’s disease [5], [7], [8], [11], which was a somewhat unanticipated finding as it implied that disease was associated with greater diffusivity along the preferential orientation of white matter tracts. An alternate hypothesis to explain this phenomenon, however, was that it might relate to differential disease involvement in crossing tracts *e.g.* if tract A and B cross, but only tract B is affected by disease, then its degeneration will lead to an apparent increase in λ_1_–and greater anisotropy–because the tensor is now being influenced more exclusively by tract A [13]. To avoid these confounds, the midline corpus callosum was studied because (i) it can be directly visualised with obvious boundary limits and (ii) it is devoid of crossing fibres. It has further advantages in that it is a large structure (*cf.* the fornix, which can also be directly visualised) hence minimising partial volume effects; finally, prior knowledge in Alzheimer’s disease, indicates that one expects greater degenerative change in the splenium compared to the genu [7]; hence one can study differential disease effects in the same white matter bundle.

**Table 4:** DTI group comparisons across disease stages and linear dependence on global cognition for all patients in the splenium.

Splenium	Cross-sectional			ACE-R Linear Dependence (N = 43)
	Very mild Alzheimer’s (N = 21) vs. Control (N = 26)	Mild Alzheimer’s (N = 22) vs. Control (N = 26)	Mild Alzheimer’s (N = 22) vs. Very mild Alzheimer’s (N = 21)	
**λ_1_**	2.62*	1.19	1.54	0.15
**RD**	2.13^§^	3.07**	−0.89	−0.40**
**MD**	3.01**	2.74*	0.21	−0.21
**FA**	−1.27	−2.85*	1.4	0.44**

Group results are given as Wilcoxon rank-sum Z-statistic; statistical dependencies are given as Pearson correlation coefficient with 41 degrees of freedom.

Significance levels: ^§^0.01<P<0.05 (Alzheimer’s disease worse than controls, two-tailed); *P<0.01 (Alzheimer’s disease worse than controls, two-tailed); **P<0.05 (Alzheimer’s disease worse than controls, two-tailed, Bonferroni-corrected on n = 24; or DTI measure correlated with Alzheimer’s disease cognitive status, two-tailed, Bonferroni-corrected on n = 12).

**Figure 6:**
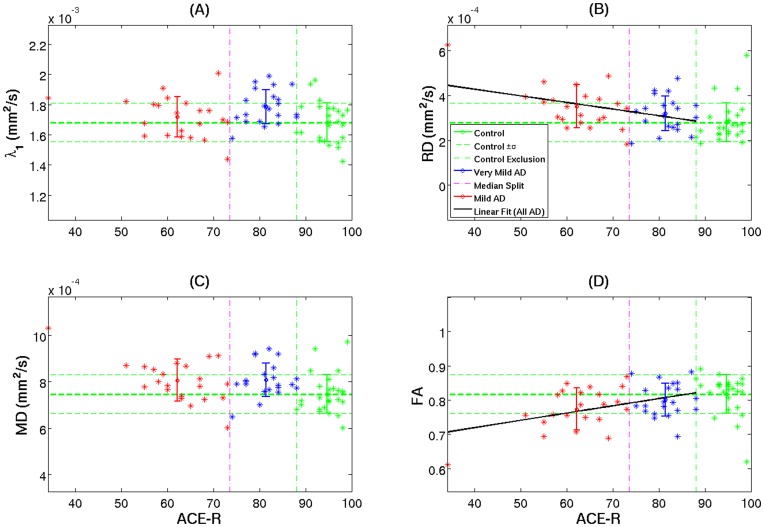
Cross-sectional diffusion tensor behaviour in the splenial region. Mean subject values for skeletonised DTI parameters in the splenium as a function of cognitive status (ACE-R scores) for controls (green), very mild Alzheimer’s disease (blue) and mild Alzheimer’s disease patients (red). The error bars represent ± one group standard deviation. The vertical axes were scaled to 10 control standard deviations. The vertical lines delimit the control exclusion criteria (ACE-R<88/100) and the median split (ACE-R = 74). A least-square linear fit was displayed if Pearson’s correlation coefficient was deemed statistically significant (Table 4).

Each T1-weighted structural volume was affine-registered to its corresponding *b*0 image, hence enabling the midline corpus callosum to be individually traced with Analyze v8.1 (Biomedical Imaging Resource, Mayo Foundation, Rochester, MN, USA), while minimising partial-volume contamination. Then in Matlab v2008a (The Mathworks Inc., Natick, MA, USA), we automated the subdivision of each corpus callosum mask into three regions–splenium, truncus and genu as illustrated in Figure 2 (top panel)–of equal length along the axis that connects the most distal–caudal and rostral–points from the mid-sagittal corpus callosum’s centre-of-mass. Note that although rigorous, tractography-based strategies have been proposed to subdivide the corpus callosum [27], in this study we were ultimately interested in discerning overall tensor differences between caudal and rostral callosal tracts; thus, for simplicity, we followed a widely-used previous classification [28]–we collapsed the isthmus and splenium regions into a single splenial region of interest, and the truncus included both anterior and posterior sections of the callosal midbody. It should also be noted that although both schemes were highly concordant in the caudal third, they disagreed on the definition of genu, which only extended across the anterior sixth of the corpus callosum in the most-recently proposed classification [27].

**Figure 7:**
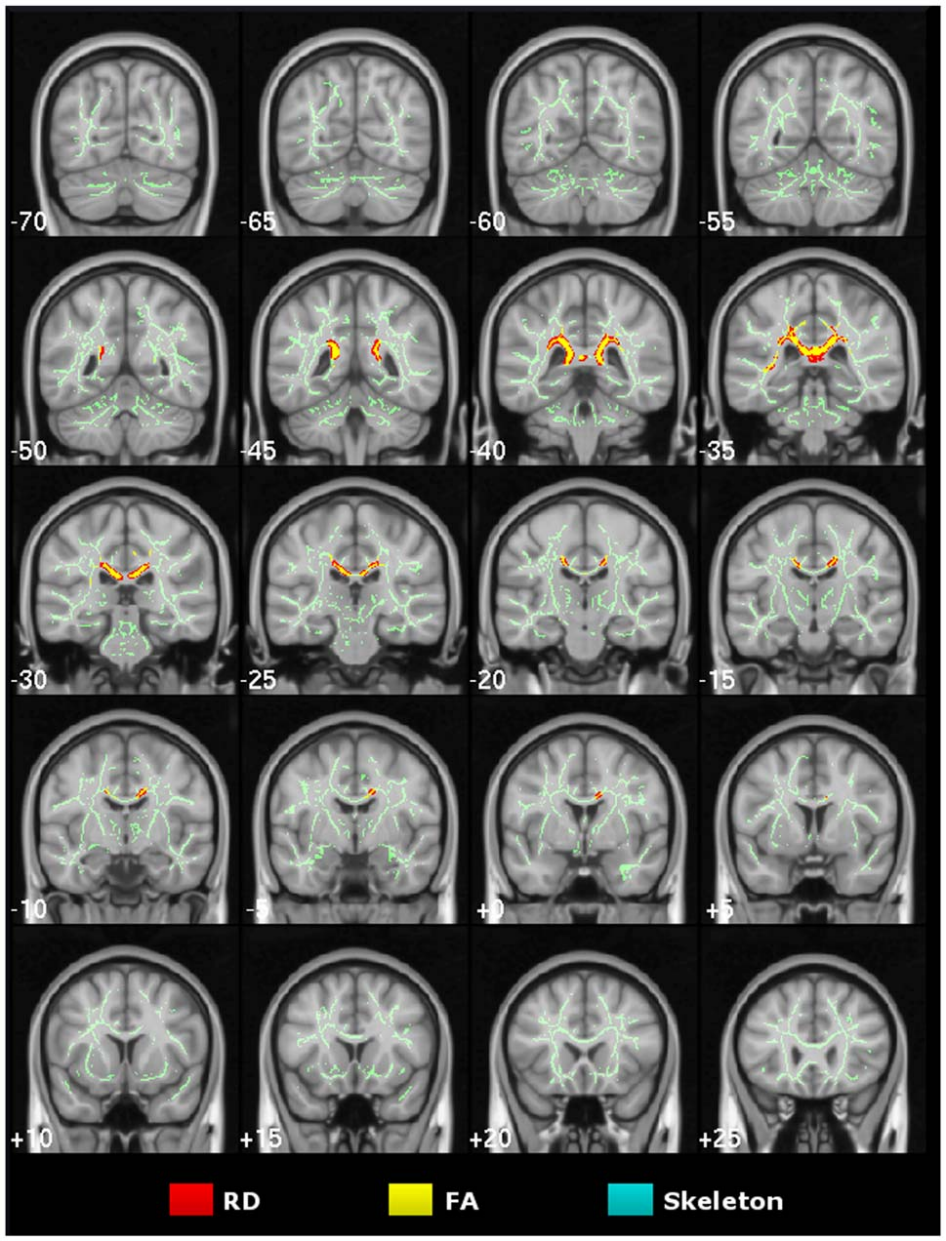
Longitudinal study of Alzheimer’s disease. Whole-brain TBSS contrast on 12-month follow-up versus baseline in the longitudinal Alzheimer’s disease cohort (TFCE-P<0.05) for increased radial diffusivity and reduced FA *n.b.* no significant results for λ_1_ or MD were found.

**Figure 8:**
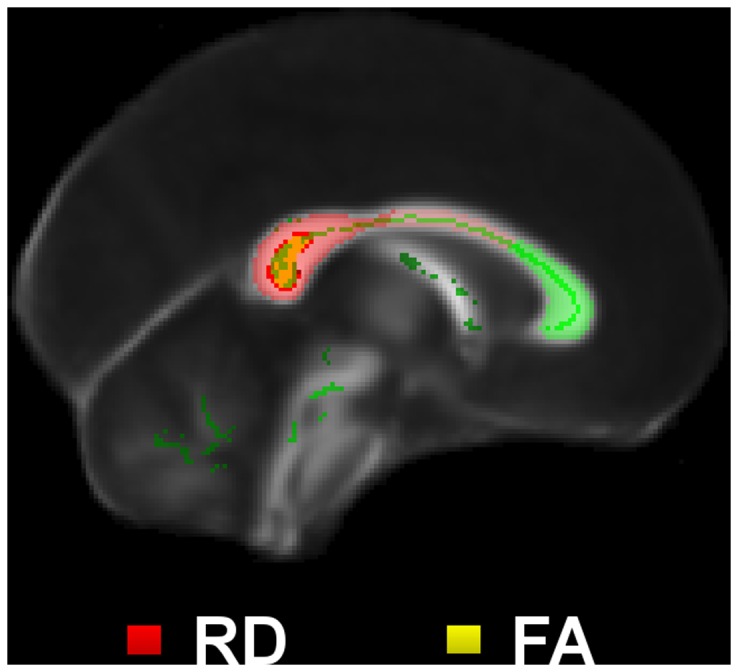
Longitudinal results in the mid-sagittal corpus callosum. Longitudinal TBSS results for radial diffusivity and fractional anisotropy across the midline.

We computed mean values for each region of interest in native space and for comparison, we also extracted mean values–directly in standard space–resulting from the intersection of each region of interest (segmented from the standard FMRIB58_FA template) with the mean FA skeleton mask (N = 69, FA>0.2) as depicted in Figure 2 (bottom panel). It is important to note that the latter method only required tracing the template corpus callosum mask once; whereas the former approach needed corpus callosum masks to be delineated for every individual. Although manual extraction is considered to be the “gold standard” method [14], it is a time-consuming process and in neurodegeneration, atrophy can lead to systematic misregistration to template of a patient group relative to controls [29]. In order to circumvent this problem, TBSS projects tract centres onto a skeleton containing all major white matter bundles; thereby minimising the effect of misregistration, but also excluding peripheral white matter tracts. In this study, we also tested the hypothesis that skeletonised mid-sagittal corpus callosum DTI data contains all the relevant information needed both to detect early white matter damage in Alzheimer’s disease and to monitor disease progression.

**Table 5:** Longitudinal DTI assessment of Alzheimer's disease in the splenium.

Splenium			Longitudinal
	Baseline (N = 16) vs.Control (N = 26)	12 months (N = 16) vs.Control (N = 26)	12 months (N = 16) vs.Baseline (N = 16)
**λ_1_**	2.37^§^	1.54	−0.52
**RD**	1.72	2.97**	−2.59*
**MD**	2.47^§^	2.71*	−1.76
**FA**	−0.87	−2.58*	−2.64*

Baseline and 12 months versus controls comparisons are reported as Wilcoxon rank-sum Z-statistic values; paired longitudinal results are given as Wilcoxon signed-rank Z-statistic.

Significance levels: ^§^0.01<P<0.05 (Alzheimer’s disease worse than controls, two-tailed); *P<0.01 (Alzheimer’s disease worse than controls; or 12 months worse than baseline, two-tailed); **P<0.05 (Alzheimer’s disease worse than controls, two-tailed, Bonferroni-corrected on n = 12).

To address the research questions posed in this study, we first collapsed all DTI measurements from all Alzheimer’s disease subjects (N = 43), which, for each callosal subregion, were cross-sectionally compared against control data (N = 26) and regressed against a measure of global cognitive status (ACE-R scores). These tests aimed to find differential behaviours across regions. Subsequently, the patient group was subdivided–as proposed in the ‘Subjects’ subsection (very mild, N = 21; and mild Alzheimer’s disease, N = 22)–, and further cross-sectional comparisons were performed to assess the overall trend of each DTI parameter across disease severity. The group study designs were as follows: both very mild and mild patient groups were contrasted against controls; additionally, mild patients were compared against the very mild cohort. Further confirmation of the observed tensor behaviour was sought by testing a smaller group of Alzheimer’s patients (N = 16) longitudinally: 12 months versus baseline, and contrasting data from both time-points against controls.

**Figure 9:**
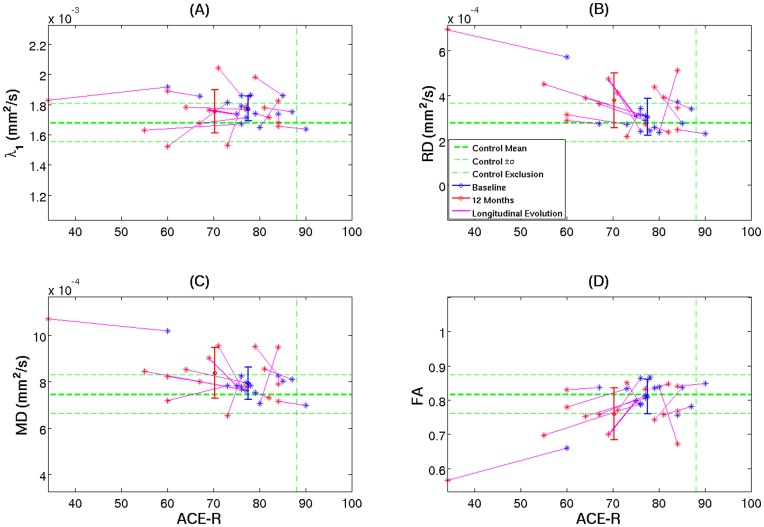
Longitudinal tensor behaviour in the splenium. Longitudinal pairs of mean subject skeletonised DTI parameters as a function of cognitive status (ACE-R) for Alzheimer’s disease subjects at baseline (blue) and 12 months (red).

In Matlab, we first applied a numerical Lilliefors test of the default null hypothesis that mean-subject values came from a distribution in the normal family [30], which revealed that they did not at α = 0.05 for any DTI metric in any cohort. We therefore compared unpaired DTI-derived values from independent samples (*i.e.* patients versus controls or very mild versus mild patients) using nonparametric Wilcoxon rank-sum–*i.e.* Mann–Whitney *U*–tests [31], [32], and paired longitudinal samples (*i.e.* same patient cohort at different time-points) were tested with the Wilcoxon signed-rank approach [32]. For consistency, all two-sample hypotheses on cognitive profiles were tested with the same relevant (*i.e.* rank-sum if cross-sectional; signed-rank if longitudinal) method. Linear dependence with ACE-R was tested using pairwise Pearson’s correlations [33]. Note that statistical significances were computed combining both tails of the sampling distributions.

To assess for atrophy in the midline corpus callosum, we compared cross-sectional callosal areas in native space. Each measurement was normalised for differences in total intracranial volume using an analysis of covariance approach, where the measured areas were adjusted by an amount proportional to the difference between each individual's observed total intracranial volume and the global total intracranial volume mean for all control subjects [34]. Total brain volumes were also computed and normalised (by total intracranial volume) to compare (N = 43) mid-sagittal callosal areas with a measure of global atrophy. Total intracranial and brain volumes were determined using a previously validated method that involved summing grey matter, white matter and cerebrospinal-fluid tissue segments [35].

#### Simultaneous inference procedure

In order to correct for the statistical effect of simultaneous regional testing in the present study, we applied Bonferroni inequalities to each family of hypotheses with α = 0.05. We corrected inferences separately if the hypotheses under evaluation were different. For instance, we performed 28 cross-sectional statistical tests to identify the nature of cognitive deficits across groups at differing disease stages relative to controls. This experiment was treated as a family (n = 28 tests); the family-wise error (FWE) rate associated with α = 0.05 was therefore P_FWE_ = 0.0018. Analogously, statistical significance (corrected for multiple comparisons) on longitudinal comparisons of cognitive performance was established at P<0.0071 (n = 7). The cross-sectional analyses (N = 43 patients versus controls)–performed to characterise the different imaging parameters in each callosal subdivision–were treated as a separate family (n = 12, P_FWE_ = 0.0042). Pearson’s correlations were also treated as a family (n = 12, P_FWE_ = 0.0042). In addition, the cross-sectional assessments of each patient subgroup (*i.e.* very mild and mild Alzheimer’s disease versus controls) were corrected together (n = 24, P_FWE_ = 0.0021). Note though that for each region of interest, longitudinal DTI data was corrected separately (n = 12, P_FWE_ = 0.0042).

## Results

### Cross-sectional TBSS Study of Very Mild Alzheimer’s Disease

TBSS results for the voxel-wise group contrast of very mild Alzheimer’s disease versus controls are shown in Figure 3. The λ_1_ statistical map showed significant bilateral and confluent change that predominantly involved parietal white matter, with strongest abnormalities along the posterior cingulum and the inter-hemispheric tracts of the caudal corpus callosum. In contrast, there was relative sparing of caudal occipital, rostral temporal lobe and prefrontal white matter, and, the more rostral tracts of the corpus callosum. RD abnormalities spatially overlapped with those found for λ_1_ in parietal and superior temporal white matter–though only across the right hemisphere. FA changes were similarly distributed, but overall were the least widespread.

### Cross-sectional TBSS Study of Mild Alzheimer’s Disease

Extensive, mostly bilateral distributions of DTI abnormalities for all metrics (increased diffusivities and reduced FA) were found in the mild Alzheimer’s disease versus control group contrast using TBSS. As illustrated in Figure 4, λ_1_ showed the most extensive and confluent clusters of significance. These were located in parietal white matter regions including the caudal corpus callosum and the posterior cingulum bundle; caudal temporal areas with a slightly greater predilection for the left side and bilateral frontal lobe involvement Although RD and FA abnormalities were highly concordant and largely overlapped with λ_1_ clusters, overall they were less extensive; in particular, frontal lobe involvement of RD and FA was minimal. Note that all regions found to be abnormal in the very mild Alzheimer’s disease group (Figure 3), were also clearly damaged in the mild group (Figure 4).

The cross-sectional MD results for both Alzheimer’s disease groups were also extensive and unsurprisingly overlapped to a high degree with those of its components; more specifically with the spatial distribution of increased λ_1_, but it did not reveal any additional involvement (see Figure S1).

### Mid-sagittal Corpus Callosum

#### TBSS

Unlike total brain volume in Alzheimer’s disease, which was significantly reduced compared to controls (standard score, Z = −0.80; P = 0.002), atrophy in the cross-sectional area of the corpus callosum did not reach statistical significance (Z = −0.43, P = 0.1).

Figure 5 shows DTI abnormalities across the mid-sagittal corpus callosum for both very mild and mild Alzheimer’s disease stages. The caudal corpus callosum was heavily involved, whereas there was preservation of white matter tracts running across the mid-sagittal genu. λ_1_ featured prominently in the very mild Alzheimer’s disease cohort but it did not become more extensive in the more-impaired group; in contrast, RD/FA abnormalities were more extensive in mild-stage Alzheimer’s disease. Note that the fornix was abnormal for all DTI metrics in both Alzheimer’s disease groups (uncorrected-P<0.01, data not shown), but the clusters of significance did not survive our attempt to correct for multiple comparisons.

### Regions of Interest

The regional analyses were consistent with the above TBSS observations. Table 3 highlights the preservation of the midline truncus and genu relative to the splenium when contrasting all Alzheimer’s patients (N = 43) with the control group. Examining for differential behaviour of the diffusion tensor across varying disease stages reinforced the finding that in early disease, λ_1_ is more prominently abnormal than RD indicating that a significant contributor to early MD increase is an increase in λ_1_ (Table 4). RD and FA, however, showed the most prominent alterations in the more cognitively-impaired group of patients–where λ_1_ remained relatively stable–, suggesting that MD abnormalities in later disease stages are primarily driven by increased RD (Table 4). The latter finding was supported by a statistically significant correlation between dementia severity (ACE-R score) and either FA or RD (Table 4). It should be noted that statistical tests for the truncus and genu–equivalent to those reported in Table 4 for the splenium–yielded, as expected, no significant results.

Focusing on the splenial lesion, this being the key pathological region, Figure 6 illustrates all mean patient DTI values relative to control data as a function of cognitive status. As shown in Table 4, splenial λ_1_ appeared to increase significantly in very mild subjects, but it did not follow that more impaired patients were increasingly abnormal–if anything there was a trend to attenuation of abnormality with advancing dementia (Figure 6a). In stark contrast, RD and FA were essentially less sensitive in very mild disease but there was a linear progression observed for these behaviours with advancing severity (Figure 6b and 6d). Note that the behaviour of MD followed a similar pattern to that of λ_1_ in early disease stages but appeared to be largely driven by RD in more advanced cases (Figure 6c).

It should be noted that the regional results reported in this section–using skeletonised DTI data–were replicated with data extracted from entire callosal regions of interest in native space (see plots in Figure S2).

### Longitudinal Study of Alzheimer’s Disease

The contrast of 12-month follow-up scans with baseline in the longitudinal Alzheimer’s disease cohort (N = 16) found that λ_1_ remained unchanged in the whole brain’s skeletonised white matter at TFCE-P<0.05. However, consistent with the cross-sectional analyses, RD/FA abnormalities progressed significantly in the caudal corpus callosum and posterior cingulum bilaterally, and in a left superior temporal region (Figure 7). The clusters of significance found in the longitudinal assessments were highly co-localised with the strongest DTI abnormalities found in the very mild Alzheimer’s disease group relative to controls; this was also clearly illustrated in the mid-sagittal TBSS results (Figure 8).

The splenial results shown in Table 5 revealed a very consistent scenario. λ_1_ was slightly abnormal at baseline but it did not progress. RD and FA differences, however, –above the statistical threshold at baseline–showed emphatic abnormalities at 12 months and during the longitudinal time-span. MD was found to be similarly abnormal at baseline and 12 months, thus resulting in no apparent progression.

When the extracted mean DTI values from each subject’s skeletonised splenium were plotted against ACE-R scores (Figure 9), the findings were in remarkable agreement with those observed in the cross-sectional study. Overall, RD/FA longitudinal pairs (Figure 9b and 9d, respectively) followed more coherently progressive evolutions than axial and mean diffusivities (Figure 9a and 9c).

## Discussion

This study aimed to investigate the biomarker potential of DTI metrics in Alzheimer’s disease through whole-brain analyses as well as by taking a reductionist approach in the corpus callosum to eliminate ambiguities generated by uncertain tract visualisation and crossing fibres. The evolution of DTI changes was examined in both cross-sectional and longitudinal datasets; the former by way of a median spilt in which 76% of the very mild patients were in the mild-cognitive impairment stage of Alzheimer’s disease, and the latter by contrasting 12-month follow-up scans to baseline. Across the various analyses, a consistent picture emerged in which an increase in λ_1_ is the first significant change in Alzheimer’s disease, but then remains relatively steady. In contrast, RD–and therefore FA–become progressively more abnormal with disease evolution, making them the candidate metrics for stage-specific biomarkers. In other words, these markers could have value to track change over time. Given that FA reduction is a function of both RD and λ_1_, and the early increase in λ_1_ appeared to attenuate with disease progression, it may be that FA is the superior measure of decline compared to RD in longitudinal studies. It should be emphasized, however, that the attenuation of λ_1_ was subtle and, unless replicated in similar studies, could simply represent random variability in the current dataset. A further caveat to this proposal is that the preference for FA would only apply to areas in which the λ_1_ abnormality has already peaked–such as the splenium in the present analyses. For instance, in a repeated-measure longitudinal design, in areas that are initially spared in terms of λ_1_, the first movement of this metric would be to increase, leading to a loss of sensitivity for FA to detect change. This was exemplified by the cross-sectional results, in which frontal and left temporal white matter was normal in the very mild group, but became abnormal (mostly in terms of λ_1_) in the mild group. This problem would, of course, be avoided by studying RD, which–unlike FA–is independent of λ_1_.

In contrast to RD and FA, the results indicated that λ_1_ has no role at all as staging biomarker for a given region of interest. Although one could, in theory, propose it as a staging marker by looking at the differential spatial extent of λ_1_ abnormalities over time, this would be difficult to implement as a marker of progression given that some degree of heterogeneity would be expected across subjects; furthermore, such an approach would also be confounded if it proved correct in future studies that the λ_1_ effect attenuates in early affected regions. Nevertheless, the results of both cross-sectional and longitudinal studies clearly indicate that an increase in λ_1_ is the first sign of change in Alzheimer’s disease, suggesting therefore that it could have a role as an early state-specific marker. This was particular notable in the cross-sectional data, in which posterior temporo-parietal white matter including the splenium showed increased λ_1_ in the very mild group with the same regions showing involvement of RD and FA in the mild group *i.e.* at a more advanced disease stage. In the more advanced group, however, new areas–notably frontal and left temporal white matter–showed increased λ_1_, suggesting that as degeneration spreads to new areas, an increase in λ_1_ is the first sign of this involvement. Similarly, the longitudinal data also showed that there was a prominent increase in λ_1_ in posterior temporo-parietal white matter at baseline (cross-sectional comparison against the control population not shown), with RD and FA abnormalities emerging in the same spatial distribution with follow-up.

Increased λ_1_ as found in this and other recent studies [5], [7] was an unanticipated finding in Alzheimer’s disease and its mechanism is uncertain. The finding that the λ_1_ abnormalities were the most spatially extensive extends the results of our earlier study using the TBSS method [5], and are also consistent with a recent independent TBSS study in showing that this metric has the greatest sensitivity to change in early disease [36]. Using a similar TBSS-based approach, Huang et al. [7] also found that the most prominent features in less affected areas in Alzheimer’s disease were increased λ_1_ and MD whereas, also consistent with our findings, in areas such as the cingulum and fornix that would be expected to have most advanced pathology, RD and FA were highly abnormal but λ_1_ was not.

It was recently proposed that λ_1_ increase might represent differential tract involvement in areas of crossing white matter tracts [13]–*i.e.* if two tracts cross then the tensor in such areas will be the average contribution of both; therefore, if one of these tracts degenerates, the tensor will be driven by the remaining tract and hence an increase in λ_1_ could be predicted. While this seems a very plausible theory in crossing fibres, it cannot explain the λ_1_ increase in very early Alzheimer’s disease because the observation is equally apparent in areas such as the splenium [5], [7] and fornix [5], [37], in which crossing tracts are not present. One clue from the current study was the finding that increased λ_1_ in the splenium showed no correlation with global dementia severity. This, in turn, implies that increased λ_1_ might be completely independent of axonal loss. It must be stressed, nevertheless, that the failure of λ_1_ to correlate with dementia severity is only demonstrated at a global level. It may still be the case that the phenomenon driving the early increase in λ_1_ is associated with local dysfunction of specific cognitive processes. For instance, a recent study noted correlation between increased λ_1_ and route-learning impairment in Alzheimer’s disease [38]; the area of correlation was tightly co-incident with correlations between the same task and both metabolism and atrophy of adjacent grey matter suggesting that it was not an artefactual result.

Studies in Alzheimer’s disease, to date, have mostly tended to conceptualise DTI abnormalities in terms of neuronal loss or damage, but it is important to keep in mind that although robust tensor changes can be found in Alzheimer’s disease, their precise underlying mechanism is essentially unknown. Furthermore, studies often infer mechanisms by citing homology with animal models, but this can only ever be *consistent with* rather than *proof of* a given mechanism; for instance, if myelin loss in an animal model causes a particular tensor metric to change in a certain way, it does not follow that myelin loss is the *only* mechanism that could generate such a tensor behaviour. Returning to the present findings, RD, and therefore FA, correlated with dementia severity implying that whatever these metrics capture, it is likely to be related to neuronal loss. The fact that increased λ_1_ was both independent of dementia severity and a precursor to RD/FA changes (*n.b.* areas where RD and FA changes emerged were characteristically those that had first seen an increase in λ_1_), suggests that λ_1_ may be capturing an upstream event to axonal degeneration. It is also noteworthy that the spread of λ_1_ increase was not random but, rather, closely mirrored the expected progression of degeneration as would be measured by cerebral glucose metabolism–*i.e.* posterior (posterior cingulate/precuneus spreading out to lateral temporo-parietal association tracts), then later to anterior (prefrontal) areas [39], [40].

Although a completely speculative hypothesis, one possible upstream event could be inflammation. The role of inflammatory change in Alzheimer’s disease is presently of considerable interest [41] and it is at least conceivable, given that λ_1_ increase appears to represent something other than axonal loss, that it could be driven by factors such as microglial activation. Whatever its mechanism, understanding the true sequence in the cascade of events leading to neuronal loss in Alzheimer’s disease is arguably the most important question in understanding pathogenesis. As such, further work to understand what this early λ_1_ increase might represent in areas that subsequently degenerate, seems to be, therefore, critically important.

The differing behaviour of tensor metrics identified in the course of Alzheimer’s disease in this study goes some way to explain inconsistencies reported in previous studies. For instance, some studies in early disease have reported little abnormality in FA [5], [6]. That said, it is difficult to identify a coherent picture from previous DTI results in Alzheimer’s disease, even if one only considers the corpus callosum–for review see [42]. Factors contributing to this inconsistency likely include: differing levels of dementia severity; an almost universal lack of outcome data in cohorts labelled mild-cognitive impairment; variable region-of-interest placement; and variable acquisition protocols. In the present study, the cross-sectional and longitudinal data highlighted the critical effect of dementia severity, while clinical follow-up ensured that all cases scanned in the mild-cognitive impairment stage had incipient Alzheimer’s disease. The issue of region-of-interest placement is critical to standardisation if DTI data is to be used as a biomarker. Because TBSS fits tract centres common to all subjects, this appears to be an appropriate method for whole-brain analyses. Meanwhile, the midline corpus callosum offers a cleaner target to study a tract in isolation; critically, however, as would be expected from prior knowledge, it is not homogenously affected but, rather, the splenium is the site of maximum damage. The current study offers an automated method for extracting corpus callosum regions that could go some way to optimising comparability across studies. Finally, the issue of acquisition is critical to consider. The present study used a 63-direction acquisition with one b-value, and as already mentioned, yielded a spread of abnormalities with advancing severity that is highly consistent with the evolution of Alzheimer’s disease from other imaging modalities. Recent methods work has validated the use of 30 directions with two b-values as an alternative, whereas fewer (than 30) directions may be less reliable for tensor modelling unless a large number of b-values are used [43]; to this end, it is important to note that most early DTI studies employed less than 10 directions with only one b-value, which may have contributed to noisy or inconsistent results. It should also be noted that at present, although RD, MD and FA are considered to be reliable DTI metrics, conflicting results have been reported with regard to the intra-scanner stability of λ_1_ measurements at 3T [44], [45]. In this study, λ_1_ appeared to be robust in the corpus callosum, but further test-retest validations using the proposed experimental methodology are required to address this important question. A final technical point to note is that although it is discouraged to interpret primary diffusivities on the basis of the underlying neurobiological processes [46], the negligible effects of: (i) crossing fibres, (ii) partial volume–due to lack of atrophy–and (iii) eigenvector sorting bias–due to the large differences in signal-to-noise ratio between λ_1_ and RD *e.g.* λ_1_ ≈ 6·RD in the skeletonised splenium–, makes the mid-sagittal corpus callosum a suitable white matter structure to monitor early-stage Alzheimer’s disease.

In conclusion, the cross-sectional and longitudinal data examined in the present study identified that RD and FA have utility as staging biomarkers in Alzheimer’s disease. Both whole-brain TBSS and a skeletonised splenial region of interest appear to be good methods to standardise sampling in repeated-measures designs. The sensitivity of these measures compared to other biomarkers needs to be established, however, one final point argues for their potential added value in practical terms. Structural MRI is already a standard procedure in longitudinal studies; a diffusion sequence can be readily acquired in the same scanning session, meaning that this added value can be achieved with little extra cost or inconvenience compared to nuclear medicine imaging or cerebrospinal-fluid analysis. Axial diffusivity (λ_1_) in a given region is a state-, rather than a stage-specific biomarker, that predates changes in RD/FA and appears to represent something other than axonal loss.

## Supporting Information

Figure S1 **Increased mean diffusivity results for very mild and mild Alzheimer’s disease.** TBSS results for very mild- and mild-stage Alzheimer’s disease groups compared to controls. Thresholded (TFCE-P<0.05) statistical maps for increased mean diffusivity were overlaid onto the mean FA skeleton and the MNI152 template with coronal depths given in millimetres. Extensive, mostly bilateral distributions of DTI abnormalities for increased mean diffusion were found in the very mild and mild Alzheimer’s disease group comparisons. Significant abnormalities were located in parietal white matter regions including the caudal corpus callosum and the posterior cingulum bundle, and in caudal temporal areas. All clusters of significance found in the very mild Alzheimer’s disease group, were also found in the mild group. As expected, overall MD abnormalities were highly concordant and largely overlapped with the spatial distribution of λ_1_ clusters of significance shown in Figure 3 and Figure 4 (main manuscript).
(TIF)

Figure S2 **Regional analysis of the splenium in native space.** Mean subject values for DTI parameters in the native splenial region as a function of cognitive status (ACE-R) for controls (green), very mild Alzheimer’s disease (blue) and mild Alzheimer’s disease patients (red). The error bars represent ± one group standard deviation, and the vertical axes were scaled to 10 control standard deviations. The vertical lines delimit the control exclusion criteria (ACE-R<88) and the median split (ACE-R = 74). A least-square linear fit was displayed if Pearson’s correlation coefficient was deemed statistically significant for N = 43 patients. In agreement with the skeletonised data shown in Figure 6 (main manuscript), λ_1_ appeared to increase most significantly in very mild subjects, whereas RD and FA followed a linear progression with advancing disease severity.
(TIF)

## References

[1] Jack CR Jr, Petersen RC, Xu YC, O’Brien PC, Smith GE, et al (1999) Prediction of AD with MRI-based hippocampal volume in mild cognitive impairment. Neurology 52: 1397–1403. 10227624 10.1212/wnl.52.7.1397 PMC2730146

[2] Fellgiebel A, Schermuly I, Gerhard A, Keller I, Albrecht J, et al (2008) Functional relevant loss of long association fibre tracts integrity in early Alzheimer’s disease. Neuropsychologia 46: 1698–1706. 18243252 10.1016/j.neuropsychologia.2007.12.010

[3] Mielke MM, Kozauer NA, Chan KC, George M, Toroney J, et al (2009) Regionally-specific diffusion tensor imaging in mild cognitive impairment and Alzheimer’s disease. Neuroimage 46: 47–55. 19457371 10.1016/j.neuroimage.2009.01.054 PMC2688089

[4] Takahashi S, Yonezawa H, Takahashi J, Kudo M, Inoue T, et al (2002) Selective reduction of diffusion anisotropy in white matter of Alzheimer disease brains measured by 3.0 Tesla magnetic resonance imaging. Neurosci Lett 332: 45–48. 12377381 10.1016/s0304-3940(02)00914-x

[5] Acosta-Cabronero J, Williams GB, Pengas G, Nestor PJ (2010) Absolute diffusivities define the landscape of white matter degeneration in Alzheimer’s disease. Brain 133: 529–539. 19914928 10.1093/brain/awp257

[6] Fellgiebel A, Wille P, Muller MJ, Winterer G, Scheurich A, et al (2004) Ultrastructural hippocampal and white matter alterations in mild cognitive impairment: a diffusion tensor imaging study. Dement Geriatr Cogn Disord 18: 101–108. 15087585 10.1159/000077817

[7] Huang H, Fan X, Weiner M, Martin-Cook K, Xiao G, et al (2012) Distinctive disruption patterns of white matter tracts in Alzheimer’s disease with full diffusion tensor characterization. Neurobiol Aging 33: 2029–2045. 21872362 10.1016/j.neurobiolaging.2011.06.027 PMC3227739

[8] Shu N, Wang Z, Qi Z, Li K, He Y (2011) Multiple diffusion indices reveals white matter degeneration in Alzheimer’s disease and mild cognitive impairment: a tract-based spatial statistics study. J Alzheimers Dis 26 Suppl 3275–285. 21971467 10.3233/JAD-2011-0024

[9] Zhang Y, Schuff N, Du AT, Rosen HJ, Kramer JH, et al (2009) White matter damage in frontotemporal dementia and Alzheimer’s disease measured by diffusion MRI. Brain 132: 2579–2592. 19439421 10.1093/brain/awp071 PMC2732263

[10] Huang J, Auchus AP (2007) Diffusion tensor imaging of normal appearing white matter and its correlation with cognitive functioning in mild cognitive impairment and Alzheimer’s disease. Ann N Y Acad Sci 1097: 259–264. 17413027 10.1196/annals.1379.021

[11] Bosch B, Arenaza-Urquijo EM, Rami L, Sala-Llonch R, Junque C, et al (2012) Multiple DTI index analysis in normal aging, amnestic MCI and AD. Relationship with neuropsychological performance. Neurobiol Aging 33: 61–74. 20371138 10.1016/j.neurobiolaging.2010.02.004

[12] Zhang Y, Schuff N, Jahng GH, Bayne W, Mori S, et al (2007) Diffusion tensor imaging of cingulum fibers in mild cognitive impairment and Alzheimer disease. Neurology 68: 13–19. 17200485 10.1212/01.wnl.0000250326.77323.01 PMC1941719

[13] Douaud G, Jbabdi S, Behrens TE, Menke RA, Gass A, et al (2011) DTI measures in crossing-fibre areas: increased diffusion anisotropy reveals early white matter alteration in MCI and mild Alzheimer’s disease. Neuroimage 55: 880–890. 21182970 10.1016/j.neuroimage.2010.12.008 PMC7116583

[14] Fellgiebel A, Muller MJ, Wille P, Dellani PR, Scheurich A, et al (2005) Color-coded diffusion-tensor-imaging of posterior cingulate fiber tracts in mild cognitive impairment. Neurobiol Aging 26: 1193–1198. 15917103 10.1016/j.neurobiolaging.2004.11.006

[15] Zhang J, Jones M, DeBoy CA, Reich DS, Farrell JA, et al (2009) Diffusion tensor magnetic resonance imaging of Wallerian degeneration in rat spinal cord after dorsal root axotomy. J Neurosci 29: 3160–3171. 19279253 10.1523/JNEUROSCI.3941-08.2009 PMC2683764

[16] Hanyu H, Asano T, Sakurai H, Imon Y, Iwamoto T, et al (1999) Diffusion-weighted and magnetization transfer imaging of the corpus callosum in Alzheimer’s disease. J Neurol Sci 167: 37–44. 10500260 10.1016/s0022-510x(99)00135-5

[17] McKhann G, Drachman D, Folstein M, Katzman R, Price D, et al (1984) Clinical diagnosis of Alzheimer’s disease: report of the NINCDS-ADRDA Work Group under the auspices of Department of Health and Human Services Task Force on Alzheimer’s Disease. Neurology 34: 939–944. 6610841 10.1212/wnl.34.7.939

[18] Folstein MF, Folstein SE, McHugh PR (1975) "Mini-mental state". A practical method for grading the cognitive state of patients for the clinician. J Psychiatr Res 12: 189–198. 1202204 10.1016/0022-3956(75)90026-6

[19] Mioshi E, Dawson K, Mitchell J, Arnold R, Hodges JR (2006) The Addenbrooke’s Cognitive Examination Revised (ACE-R): a brief cognitive test battery for dementia screening. Int J Geriatr Psychiatry 21: 1078–1085. 16977673 10.1002/gps.1610

[20] Reese TG, Heid O, Weisskoff RM, Wedeen VJ (2003) Reduction of eddy-current-induced distortion in diffusion MRI using a twice-refocused spin echo. Magn Reson Med 49: 177–182. 12509835 10.1002/mrm.10308

[21] Griswold MA, Jakob PM, Heidemann RM, Nittka M, Jellus V, et al (2002) Generalized autocalibrating partially parallel acquisitions (GRAPPA). Magn Reson Med 47: 1202–1210. 12111967 10.1002/mrm.10171

[22] Smith SM, Jenkinson M, Woolrich MW, Beckmann CF, Behrens TE, et al (2004) Advances in functional and structural MR image analysis and implementation as FSL. Neuroimage 23 Suppl 1S208–219. 15501092 10.1016/j.neuroimage.2004.07.051

[23] Jenkinson M, Smith S (2001) A global optimisation method for robust affine registration of brain images. Med Image Anal 5: 143–156. 11516708 10.1016/s1361-8415(01)00036-6

[24] Smith SM (2002) Fast robust automated brain extraction. Hum Brain Mapp 17: 143–155. 12391568 10.1002/hbm.10062 PMC6871816

[25] Smith SM, Jenkinson M, Johansen-Berg H, Rueckert D, Nichols TE, et al (2006) Tract-based spatial statistics: voxelwise analysis of multi-subject diffusion data. Neuroimage 31: 1487–1505. 16624579 10.1016/j.neuroimage.2006.02.024

[26] Smith SM, Nichols TE (2009) Threshold-free cluster enhancement: addressing problems of smoothing, threshold dependence and localisation in cluster inference. Neuroimage 44: 83–98. 18501637 10.1016/j.neuroimage.2008.03.061

[27] Hofer S, Frahm J (2006) Topography of the human corpus callosum revisited–comprehensive fiber tractography using diffusion tensor magnetic resonance imaging. Neuroimage 32: 989–994. 16854598 10.1016/j.neuroimage.2006.05.044

[28] Witelson SF (1989) Hand and sex differences in the isthmus and genu of the human corpus callosum. A postmortem morphological study. Brain 112: 799–835. 2731030 10.1093/brain/112.3.799

[29] Snook L, Plewes C, Beaulieu C (2007) Voxel based versus region of interest analysis in diffusion tensor imaging of neurodevelopment. Neuroimage 34: 243–252. 17070704 10.1016/j.neuroimage.2006.07.021

[30] Lilliefors HW (1967) On the Kolmogorov-Smirnov test for normality with mean and variance unknown. Journal of the American Statistical Association 62: 399–402.

[31] Mann HB, Whitney DR (1947) On a Test of Whether one of Two Random Variables is Stochastically Larger than the Other. Annals of Mathematical Statistics 18: 50–60.

[32] Wilcoxon F (1946) Individual comparisons of grouped data by ranking methods. J Econ Entomol 39: 269. 20983181 10.1093/jee/39.2.269

[33] Pearson K (1896) Mathematical Contributions to the Theory of Evolution. III. Regression, Heredity and Panmixia. Philosophical Transactions of the Royal Society A 187: 253–318.

[34] Jack CR Jr, Twomey CK, Zinsmeister AR, Sharbrough FW, Petersen RC, et al (1989) Anterior temporal lobes and hippocampal formations: normative volumetric measurements from MR images in young adults. Radiology 172: 549–554. 2748838 10.1148/radiology.172.2.2748838

[35] Pengas G, Pereira JM, Williams GB, Nestor PJ (2009) Comparative reliability of total intracranial volume estimation methods and the influence of atrophy in a longitudinal semantic dementia cohort. J Neuroimaging 19: 37–46. 18494772 10.1111/j.1552-6569.2008.00246.x

[36] O’Dwyer L, Lamberton F, Bokde AL, Ewers M, Faluyi YO, et al (2011) Multiple indices of diffusion identifies white matter damage in mild cognitive impairment and Alzheimer’s disease. PLoS One 6: e21745. 21738785 10.1371/journal.pone.0021745 PMC3128090

[37] Oishi K, Akhter K, Mielke M, Ceritoglu C, Zhang J, et al (2011) Multi-modal MRI analysis with disease-specific spatial filtering: initial testing to predict mild cognitive impairment patients who convert to Alzheimer’s disease. Front Neurol 2: 54. 21904533 10.3389/fneur.2011.00054 PMC3160749

[38] Pengas G, Williams GB, Acosta-Cabronero J, Ash TWJ, Hong YT, et al (2012) The relationship of topographical memory performance to regional neurodegeneration in Alzheimer’s disease. Front Ag Neurosci 4: 17. 10.3389/fnagi.2012.00017 PMC3389330 22783190

[39] Chase TN, Foster NL, Fedio P, Brooks R, Mansi L, et al (1984) Regional cortical dysfunction in Alzheimer’s disease as determined by positron emission tomography. Ann Neurol 15 Suppl: S170–174 10.1002/ana.410150732 6611118

[40] Nestor PJ, Fryer TD, Smielewski P, Hodges JR (2003) Limbic hypometabolism in Alzheimer’s disease and mild cognitive impairment. Ann Neurol 54: 343–351. 12953266 10.1002/ana.10669

[41] Wyss-Coray T, Rogers J (2012) Inflammation in Alzheimer disease-a brief review of the basic science and clinical literature. Cold Spring Harb Perspect Med 2: a006346. 22315714 10.1101/cshperspect.a006346 PMC3253025

[42] Di Paola M, Spalletta G, Caltagirone C (2010) In vivo structural neuroanatomy of corpus callosum in Alzheimer’s disease and mild cognitive impairment using different MRI techniques: a review. J Alzheimers Dis 20: 67–95. 20164572 10.3233/JAD-2010-1370

[43] Correia MM, Carpenter TA, Williams GB (2009) Looking for the optimal DTI acquisition scheme given a maximum scan time: are more b-values a waste of time? Magn Reson Imaging 27: 163–175. 18687552 10.1016/j.mri.2008.06.011

[44] Danielian LE, Iwata NK, Thomasson DM, Floeter MK (2010) Reliability of fiber tracking measurements in diffusion tensor imaging for longitudinal study. Neuroimage 49: 1572–1580. 19744567 10.1016/j.neuroimage.2009.08.062 PMC2789889

[45] Takao H, Hayashi N, Kabasawa H, Ohtomo K (2012) Effect of scanner in longitudinal diffusion tensor imaging studies. Hum Brain Mapp 33: 466–477. 21391276 10.1002/hbm.21225 PMC6869949

[46] Wheeler-Kingshott CA, Cercignani M (2009) About "axial" and "radial" diffusivities. Magn Reson Med 61: 1255–1260. 19253405 10.1002/mrm.21965

