# Supplementary figures and images for: Diffusion Tensor Metrics as Biomarkers in Alzheimer's Disease
Source: PLoS One. 2012 Nov 7;7(11):e49072. doi: 10.1371/journal.pone.0049072 (PMC3492261; doi:10.1371/journal.pone.0049072)

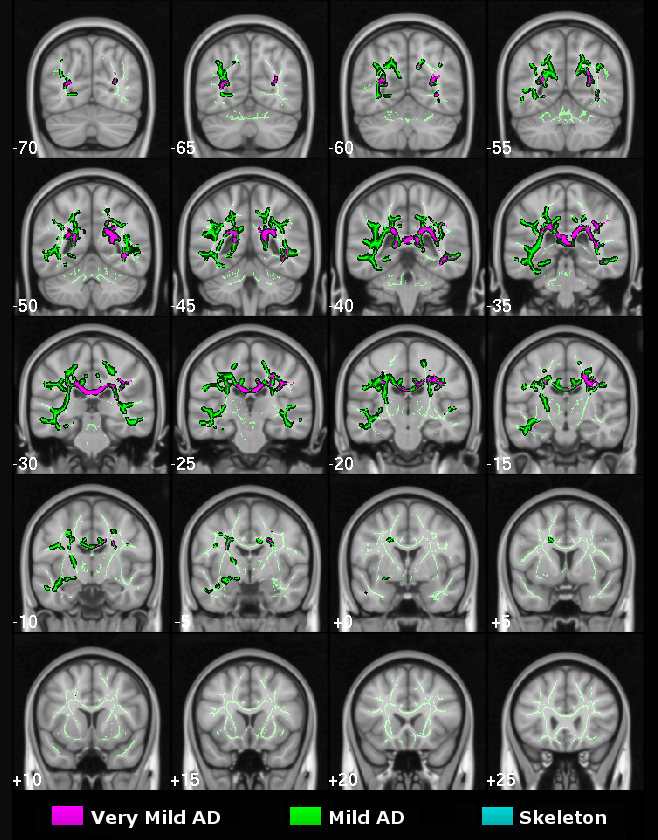

Supplement: Figure S1 — Increased mean diffusivity results for very mild and mild Alzheimer’s disease. TBSS results for very mild- and mild-stage Alzheimer’s disease groups compared to controls. Thresholded (TFCE-P<0.05) statistical maps for increased mean diffusivity were overlaid onto the mean FA skeleton and the MNI152 template with coronal depths given in millimetres. Extensive, mostly bilateral distributions of DTI abnormalities for increased mean diffusion were found in the very mild and mild Alzheimer’s disease group comparisons. Significant abnormalities were located in parietal white matter regions including the caudal corpus callosum and the posterior cingulum bundle, and in caudal temporal areas. All clusters of significance found in the very mild Alzheimer’s disease group, were also found in the mild group. As expected, overall MD abnormalities were highly concordant and largely overlapped with the spatial distribution of λ1 clusters of significance shown in Figure 3 and Figure 4 (main manuscript). (TIF) [file pone.0049072.s001.tif]

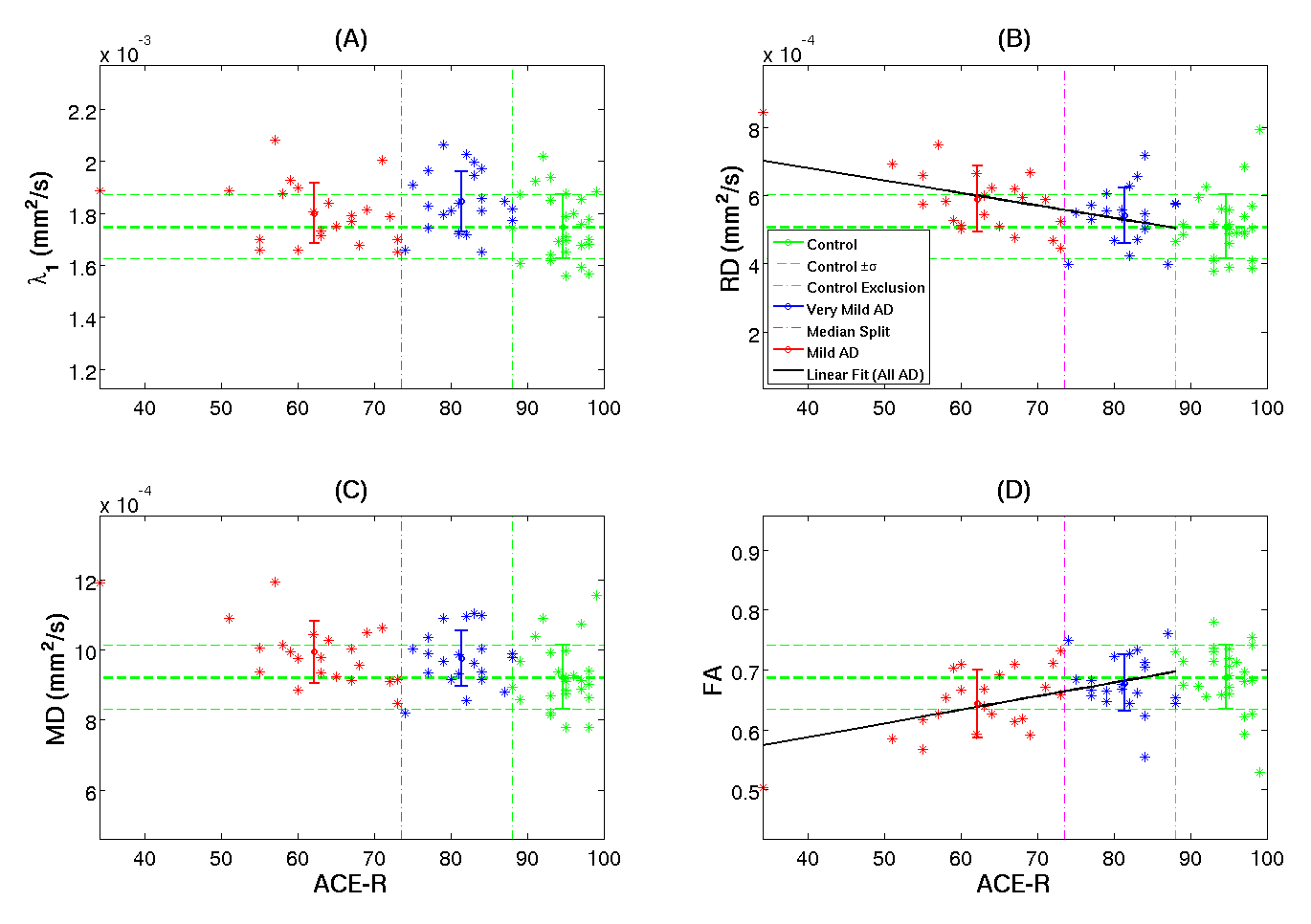

Supplement: Figure S2 — Regional analysis of the splenium in native space. Mean subject values for DTI parameters in the native splenial region as a function of cognitive status (ACE-R) for controls (green), very mild Alzheimer’s disease (blue) and mild Alzheimer’s disease patients (red). The error bars represent ± one group standard deviation, and the vertical axes were scaled to 10 control standard deviations. The vertical lines delimit the control exclusion criteria (ACE-R<88) and the median split (ACE-R = 74). A least-square linear fit was displayed if Pearson’s correlation coefficient was deemed statistically significant for N = 43 patients. In agreement with the skeletonised data shown in Figure 6 (main manuscript), λ1 appeared to increase most significantly in very mild subjects, whereas RD and FA followed a linear progression with advancing disease severity. (TIF) [file pone.0049072.s002.tif]
